# Microbiome compositional changes and clonal engraftment in a phase 3 trial of fecal microbiota, live-jslm for recurrent *Clostridioides difficile* infection

**DOI:** 10.1080/19490976.2025.2520412

**Published:** 2025-06-24

**Authors:** Josh Claypool, Gustav Lindved, Pernille Neve Myers, Tonya Ward, Henrik Bjørn Nielsen, Ken F. Blount

**Affiliations:** a Ferring Microbiome, Inc, Roseville, MN, USA; b Clinical Microbiomics A/S, Copenhagen, Denmark

**Keywords:** CDI, *Clostridioides difficile*, dysbiosis, microbiota-based product, engraftment, pharmacokinetics, pharmacodynamics, microbiome

## Abstract

Live microbiota therapies have shown promise in many gastrointestinal diseases, including in the prevention of recurrent *Clostridioides difficile* infections (rCDI); however, frameworks for their pharmacokinetic and pharmacodynamic analysis are not fully established. Fecal microbiota, live-jslm (RBL) is the first microbiota-based product approved by the US Food and Drug Administration for the prevention of rCDI and was superior to placebo in the PUNCH™ CD3 phase 3 clinical trial (NCT03244644). In this analysis, deep shotgun metagenomic sequencing was used to assess changes in gut microbiome compositions of participants and engraftment of bacterial clonal populations (i.e. strains) from RBL to recipients. Among RBL responders, gut microbiota shifted toward compositions that resembled healthy donors as early as 1 week after RBL administration; the resulting microbiota compositions included clonal populations that engrafted from RBL to recipients. Engraftment was higher in RBL responders compared with non-responders, and many clonally engrafted populations persisted for ≥ 6 months. Bacteroidia species were among the most effectively engrafted species from RBL. This study utilizes data from a large clinical trial to establish a method with high specificity for exploring clonal engraftment from microbiota-based treatments to facilitate future pharmacokinetic and pharmacodynamic analyses.

**Clinicaltrials Registration**: NCT03244644

## GRAPHICAL ABSTRACT

**Figure:**
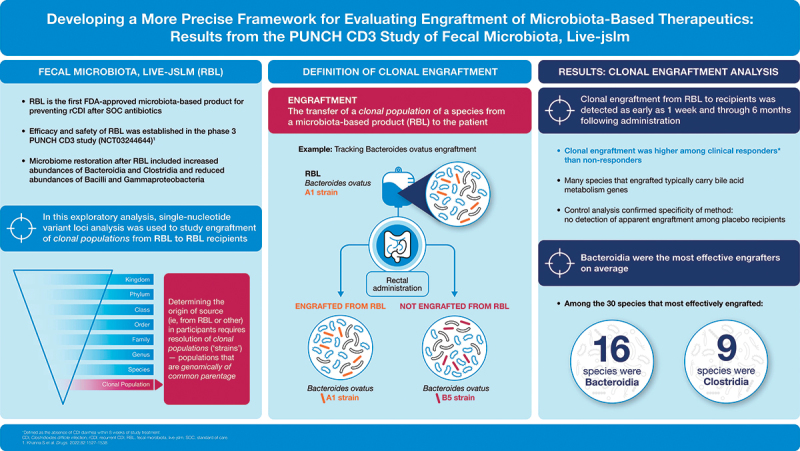

## Introduction

The human gut microbiome is a complex and diverse consortium of microorganisms that facilitate critical functions such as maintenance of the gut epithelial barrier, regulation of host immunity, and protection against opportunistic pathogens.^1,2^ In most healthy individuals, the majority of bacterial species belong to 4 major phyla,^1^ within which the classes of Bacteroidia and Clostridia predominate.^3^ The composition of the microbiome is shaped by host factors such as age,^4^ sex,^5^ health status,^6,7^ medication use,^8,9^ and diet,^10,11^ as well as environmental factors such as intestinal transit time^12^ or metabolites (e.g. bile acids^13^ and short-chain fatty acids).^14^ Significant disruption of the microbiome, sometimes termed dysbiosis, promotes gastrointestinal disease states such as *Clostridioides difficile* infection (CDI).^2^ Patients with recurrent CDI (rCDI) have reduced microbial diversity, decreased abundance of Bacteroidia and Clostridia, and increased vulnerability to *C. difficile* colonization.^15^ Microbiota-based therapeutics can reduce the risk of rCDI^16,17^ through restoration of metabolic processes such as short chain fatty acid and bile acid metabolism which impacts both the host immune system and *C. difficile* sporulation, and through competitive exclusion by filling niches and limiting access to resources, thus helping to resist *C. difficile* colonization.^18,19^ Investigations of the mechanisms for these microbiota-based products have mostly focused on microbiome or metabolome compositional changes in patients, with less focus on which bacterial populations transfer from therapeutic to recipients and then propagate – a process termed *engraftment* that was adopted from stem cell transplantation.^20^ Although a few studies have looked at engraftment after administration of microbial biotherapeutics, they have analyzed only a small number of recipients and doses, or have described engraftment with limited taxonomic resolution.^21,22^

Fecal microbiota, live-jslm (REBYOTA®; RBL, previously known as RBX2660), is the first single-dose, microbiota-based product approved by the US Food and Drug Administration (FDA) to prevent rCDI in adults following standard of care antibiotic treatment.^23^ RBL is manufactured under standardized good manufacturing practices, including rigorous donor screening and pathogen testing, to help ensure patient safety and avoid potential risks typically associated with fecal microbiota transplantation (FMT).^24^ In a randomized, placebo-controlled, phase 3 study (PUNCH^TM^ CD3; NCT03244644), RBL was superior to placebo in preventing rCDI,^16^ and restorative shifts in participants’ gut microbiome and metabolome compositions were detected as early as 1 week after RBL administration and sustained for ≥6 months.^25^ This exploratory analysis aims to investigate the extent to which these previously described microbiome and metabolome restoration changes are attributable to engraftment of clonal populations (i.e. strains) from RBL to recipients. In this context, a clonal population is defined as a population that is genomically indistinguishable or so similar that it is presumed to be from common parentage.^26,27^ The correlation of engraftment with clinical efficacy and patient covariates was also assessed, as was the relative engraftment effectiveness of different species. Collectively, these analyses aim to advance the tools and framework for understanding pharmacokinetics (PK) and pharmacodynamics (PD) for microbiota-based biotherapeutics.

## Materials and methods

### PUNCH^TM^ CD3 trial design

PUNCH^TM^ CD3 (NCT03244644) was a prospective, multicenter, randomized, double-blind, placebo-controlled phase 3 trial that evaluated the efficacy and safety of RBL for the prevention of rCDI and has been previously described.^16^ Participants were ≥18 years old with a diagnosis of rCDI and had either: a) ≥2 documented recurrences of CDI after a primary episode and had completed ≥ 2 rounds of standard of care oral antibiotic therapy, or b) ≥2 documented episodes of severe CDI resulting in hospitalization. Participants received antibiotics for their enrolling CDI episode before study drug administration. Major exclusion criteria were previously described and included previous FMT therapy.

### RBL administration

Screening, testing, and manufacturing of RBL are aligned with FDA recommendations and performed under good manufacturing practices as previously described.^24^ Each 150 mL dose of RBL contains between 1 × 10^8^ and 5 × 10^10^ colony-forming units/mL of fecal microbes. Participants were randomized in a 2:1 ratio to receive RBL or placebo (saline); the randomization schedule was created using random blocks with 4 strata based on antibiotic use at screening (vancomycin alone, vancomycin in combination, fidaxomicin, or other antibiotic). Eligible participants received a single dose of blinded RBL or placebo through rectal administration following an antibiotic washout period of 24 to 72 hours.^16^

### Study outcomes

The primary endpoint for PUNCH^TM^ CD3 was treatment success, defined as the absence of CDI diarrhea within 8 weeks of study treatment; participants were followed through 6 months.^16^ Key secondary outcomes included sustained clinical response, defined as treatment success of the presenting CDI recurrence at 8 weeks and no new CDI episodes through 6 months after completion of study treatment. Participants with CDI recurrence within 8 weeks of administration of RBL or placebo were eligible to receive open-label RBL within 21 calendar days of failure determination, which could be administered after CDI antibiotics per investigator discretion.

### Participant fecal sample collection

Clinical trial participants consented to voluntarily provide fecal samples. Consenting participants received kits to collect whole stool samples at home and shipped samples in a freezer pack via overnight courier to Ferring Microbiome. Requested time points for sample collection were prior to RBL or placebo administration (baseline); 1, 4, and 8 weeks; and 3 and 6 months after the blinded study drug administration. Upon receipt, samples were aliquoted and frozen at − 80°C with no added stabilizers until analysis.

### Sample sequencing and taxonomic annotation

Fecal samples were extracted using PowerSoil Pro kits (Qiagen, Germantown, MD) and subjected to deep shotgun sequencing (median 29 million paired-end reads per sample) (Diversigen, Minneapolis, MN). Raw FASTQ files were filtered to remove host contamination by discarding read pairs if either read mapped to the human reference genome GRCh38.p14 with Bowtie2 (v. 2.4.2).^28^ Reads were then trimmed to remove adapters and bases with a Phred score below 30 using AdapterRemoval (v. 2.3.1).^29^ Read pairs in which both reads passed filtering with a length of ≥ 100 base pairs (bp) were retained; these were classified as high-quality non-host (HQNH) reads. HQNH reads were mapped to the Clinical Microbiomics Human Microbiome Profiler (CHAMP) gene catalog using BWA mem (v. 0.7.17).^30^ An individual read was considered uniquely mapped to a gene if the mapping quality (MAPQ) was ≥ 20 and the read aligned with ≥ 95% identity over ≥ 95 bp. Per-sample species abundances were determined by the number of reads mapped to a set of species-specific genes that were previously optimized for accurate abundance profiling of each species in the CHAMP catalog, without normalization or rarefaction.^31^ Rarefied species abundance profiles, which were used for diversity and number of new species analyses, were calculated by random sampling without replacement, of 6183 signature gene counts per sample.

### Determination of clonal engraftment

All species for each administered RBL dose were compared pairwise to those found in participants who received that RBL dose. For any species found in both samples, the origin (RBL or non-RBL) for the clonal population in participants was determined based on similarity across a minimum of 50 species-specific polymorphic positions (PMPs), defined as all the positions in a species where ≥ 2 different alleles were observed in the total sample population of all RBL doses and participant baseline samples, for each participant. Alleles required a coverage of ≥ 2 reads in a sample, and, for each polymorphic position, the allele frequency within the specific sample must have been > 98% among the total population of per-participant baseline and all measured RBL samples to be considered for inclusion. It was assumed that baseline clonal populations differed among participants and were different from RBL samples, while strains from multiple samples of the same RBL batch were the same. This approach was used to train a weighted logistic regression classifier that provided a minimum similarity cutoff for determining clonality between post-administration samples and RBL dose received. Comparisons with < 80% confidence to be classified as different clonal populations were labeled as undetermined. For species where the logistic classifier had an area under the curve of < 0.8, a PMP similarity threshold of 99% was set for clonality.

As a control analysis, all species from randomly selected RBL doses were paired with each sample from placebo recipients and overlapping PMPs were compared to assess the specificity of clonal engraftment.

### Bile acid gene profiling

Bile acid gene profiles for engrafted species were determined based on the presence or absence of *bsh*, *bai* operon genes (*baiA, baiB, baiCDH, baiEI, baiF, baiG, baiN*), and/or 7-alpha/beta-HSDH genes in the corresponding reference species from the Clinical Microbiomics CHAMP gene catalog. Annotation of these genes in the CHAMP gene catalog was based on BLAST v. 2.8.1 alignment (60% similarity and 80% coverage) of CHAMP proteins against reference protein sequences for 208 *bsh* genes from 140 species,^32,33^ 58 *bai* operon genes, thirty-one 7-alpha-HSDH genes, and five 7-beta-HSDH (described in the KEGG database https://www.genome.jp/pathway/map00121).^33^ Annotation assignments were confirmed by comparing functional domain profiles using Pfam v. 33.1.

### Calculations and statistical analyses

#### Beta diversity and non-metric multidimensional scaling (NMDS)

Non-metric multidimensional scaling (NMDS) visualization of Bray-Curtis dissimilarity was conducted within the phyloseq (v. 1.44.0) R 4.3.0 package using rarefied abundance data at the species level subset for samples only from participants with a valid time point. All participants with microbiome data and an adjudicated outcome were included in the analysis (*n* = 117 participants, 445 samples, and 97 doses).

#### Alpha diversity and newly appearing species

Alpha diversity was analyzed using rarefied abundance data within the phyloseq R package. Richness analysis was conducted using rarefied abundance data, which was converted to presence/absence data. Newly appearing species were analyzed using rarefied abundance data that was converted to presence and absence data, where the presence of baseline species was subtracted from all subsequent time points. Only participants with baseline samples were included in the analysis (*n* = 143 total participants: *n* = 94 RBL, *n* = 49 placebo). Significance was calculated using Wilcoxon rank-sum test.

#### Taxonomic composition analysis

Taxonomic composition analysis was conducted using abundance data subset to 7 classes (Bacteroidia, Clostridia, Gammaproteobacteria, Bacilli, Actinomycetia, Verrucomicrobiae and Negativicutes) as well as an “other” category. Group mean relative abundance (π) with confidence intervals was derived for each time point, treatment, and outcome group by fitting relative abundance data to a Dirichlet-multinomial function via maximum likelihood estimation^34^ using the HMP R package (v. 2.0.1).

#### Engraftment analysis

Engraftment by outcome, age, sex, antibiotic use, and prior CDI episodes used data from RBL recipients at the 1-week time point. Significance was calculated by Wilcoxon rank-sum test. Comparison of engraftment to secondary:primary bile acid ratio at the 1 week time point used previously published data collected as described.^25^

#### Fraction of species engrafted from RBL

The fraction of participant Bacteroidia or Clostridia species that were clonally engrafted from RBL was calculated for each participant and time point by dividing the number of engrafted species per class by the rarefied species richness per class.

#### Engraftment effectiveness and bile acid gene analyses

Engraftment effectiveness was calculated for each species as the number of participants in which clonal engraftment was observed in ≥ 1 sample divided by the number of RBL doses that contained that species:

$$\mathrm{Engraftment}\;\mathrm{Effectivenes}s_{\mathrm{SpeciesA}}=\frac{\mathrm{Number}\;\mathrm{of}\;\mathrm{Detected}\;\mathrm{Engraftment}s_{\mathrm{Species}\;A}}{\mathrm{Total}\;\mathrm{Number}\;\mathrm{of}\;\mathrm{Doses}\;\mathrm{Presen}t_{\mathrm{Species}\;A}}$$

Participant samples with < 10 million reads were not included in the analysis; all RBL doses had > 10 million reads. Species present at < 0.1% relative abundance were removed from the analysis since that was the estimated lower limit for accurate determination of engraftment.

#### Engraftment persistence analysis

Persistence was calculated among the subset of 33 participants who provided samples for all requested time points and was defined as the number of participants in which engraftment was detected for each species at each time point divided by the total number of participants in which engraftment of that species occurred at least once.

$$\mathrm{Engraftment}\;\mathrm{Persistanc}e_{\mathrm{Species}\;A}=\text{\textbackslash{}break}\frac{\mathrm{Number}\;\mathrm{of}\;\mathrm{Detected}\;\mathrm{Engraftment}s_{\mathrm{Species}\;A}}{\mathrm{Number}\;\mathrm{of}\;\mathrm{Participants}\;\mathrm{with}\;\mathrm{Engraftmen}t_{\mathrm{Species}\;A}}$$

Only species with clonal engraftment in ≥ 5 different participants were included. For calculation of persistence, “undetermined” engraftment based on the logistic classifier was treated as not engrafted.

#### Correlation between bile acid metabolites and engrafters

A Pearson correlation coefficient was determined between the number of species engrafted and the ratio of secondary to primary bile acids as previously measured for RBL responders and non-responders at 1 week (ggpubr, v 0.6.0).^35^

## Results

### Microbiome restoration after RBL or placebo administration

**Figure 1.**
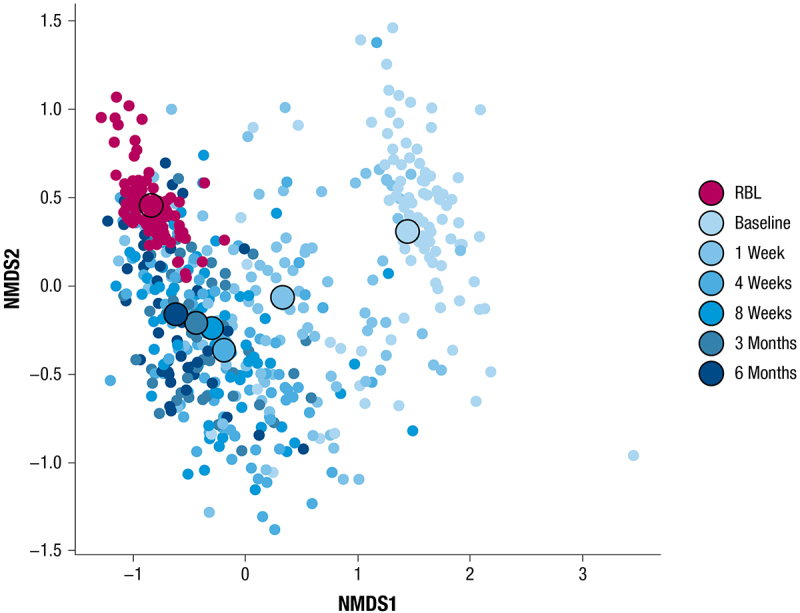
NMDS of Bray-Curtis distance calculated on species-level taxonomy of stool samples for baseline and post-RBL administration time points. The black outlined circles represent the centroid of each group or time point. NMDS, non-metric multidimensional scaling; RBL, fecal microbiota, live-jslm.

**Figure 2.**
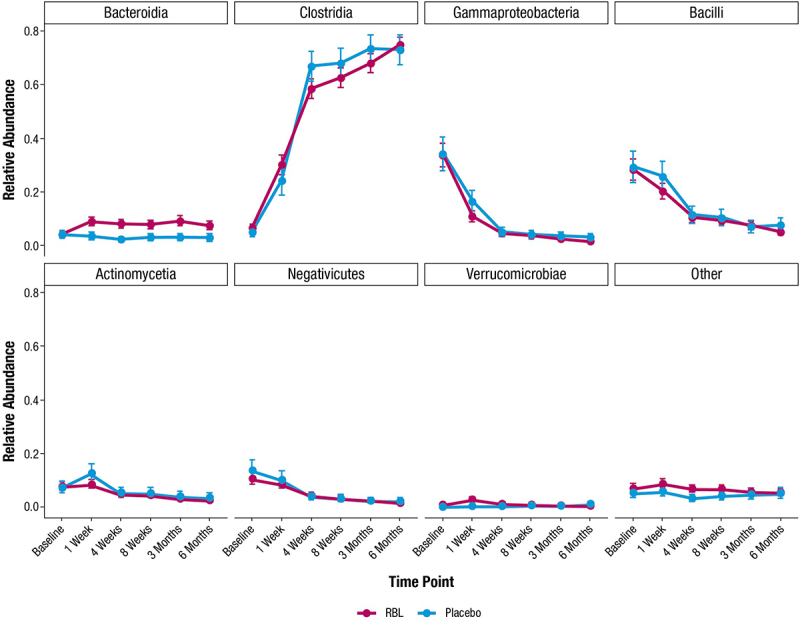
Group mean relative abundances (π) with 95% standard error for the 7 most abundant bacterial classes in participants administered RBL and placebo, based on Dirichlet multinomial. RBL, fecal microbiota, live-jslm.

Participants in PUNCH^TM^ CD3 were requested to provide fecal samples prior to administration of blinded study drug (baseline), at 1, 4, and 8 weeks, and 3 and 6 months after administration of the blinded study drug. A total of 177 participants provided ≥ 1 sample (120 RBL recipients, 57 placebo recipients), and 33 RBL recipients provided samples at all requested time points. RBL recipients’ microbiome compositions were very different from RBL compositions at baseline, but appeared to converge toward RBL as early as 1 week after RBL administration, and continued to converge closer to RBL throughout the 6 months after administration (Figure 1). Microbiome shifts were also observed among placebo recipients, although with less apparent convergence toward RBL compositions (Supplementary Figure 1). At the taxonomic class level, the shifts in both treatment arms were characterized by increased relative abundance of Bacteroidia and Clostridia after administration, concurrent with decreased relative abundance of Gammaproteobacteria, Bacilli, and Negativicutes after administration (Figure 2). As was observed in a shallow-shotgun sequencing analysis,^36^ a key difference between treatment arms was an apparent increase in Bacteroidia in RBL recipients compared to placebo recipients at all time points after administration.

**Figure 3.**
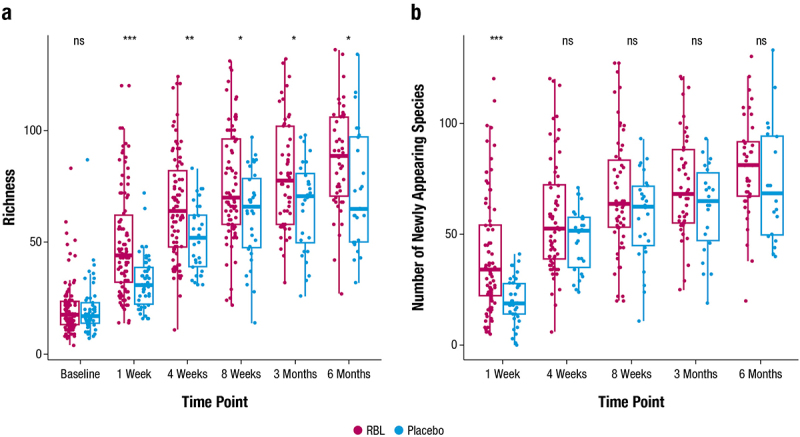
a) Species richness and b) number of newly appearing species following RBL and placebo administration. Median (thick horizontal lines) and interquartile ranges (boxes) are shown. Significance assessed via Wilcoxon rank-sum test. ns = not significant, *p < 0.05, **p < 0.01, ***p < 0.0001. RBL, fecal microbiota, live-jslm.

Species richness increased at all time points after RBL or placebo administration compared with baseline, with significantly higher richness in RBL recipients than in placebo recipients at all post-administration time points (Figure 3(a)). The Shannon diversity index also increased after administration, more so among RBL than placebo recipients at all time points except at 3 months (Supplementary  Figure 2). In both treatment arms, many of the species present after administration were not detected at baseline (newly appearing species; Figure 3(b)), underscoring large microbiome composition changes. RBL recipients had significantly more newly appearing species than placebo recipients at 1 week after administration.

### Engraftment of clonal populations from RBL

**Figure 4.**
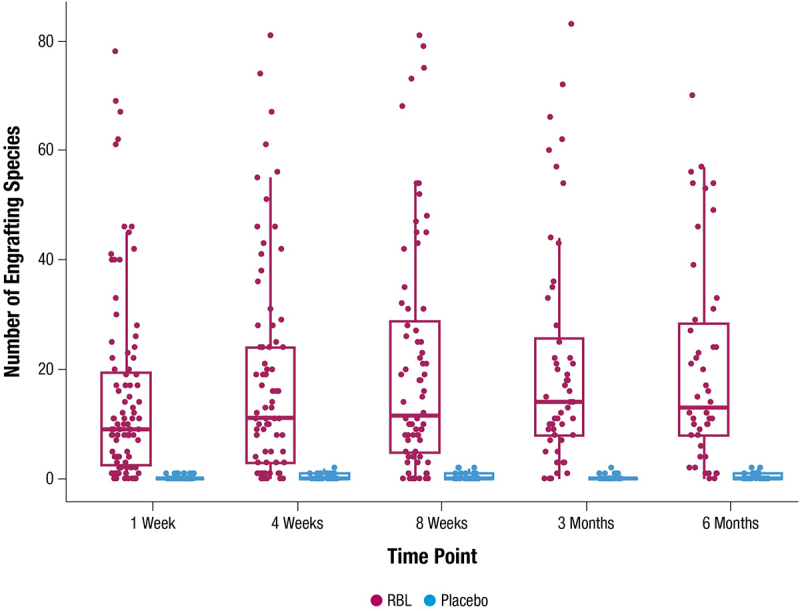
Number of engrafting species at each time point in participants administered RBL or placebo. Median (thick horizontal lines) and interquartile ranges (boxes) are shown. RBL, fecal microbiota, live-jslm.

Species detected after RBL administration were determined to have clonally engrafted from RBL if the species was also present in the RBL dose received, and if polymorphic regions associated with that species had high sequence similarity between participant sample and RBL dose. Sufficient similarity could be assessed for most species that were present at a relative abundance of ≥ 0.1% (Supplementary Figure 3). A median of 9 species per participant (range, 0 to 78) clonally engrafted from RBL to participants by 1 week after administration (Figure 4). The number of engrafting species remained similar through the monitoring period, with a median of 13 species (range, 0 to 70) engrafted at 6 months. To confirm the specificity of this method, each post-administration time point from placebo recipients was paired with a random RBL dose, and the polymorphic regions were compared among common species. Negligible polymorphic region similarity was found in these comparisons (i.e. minimal detection of apparent clonal engraftment in placebo recipients), indicating high specificity of the method (Figure 4).

**Figure 5.**
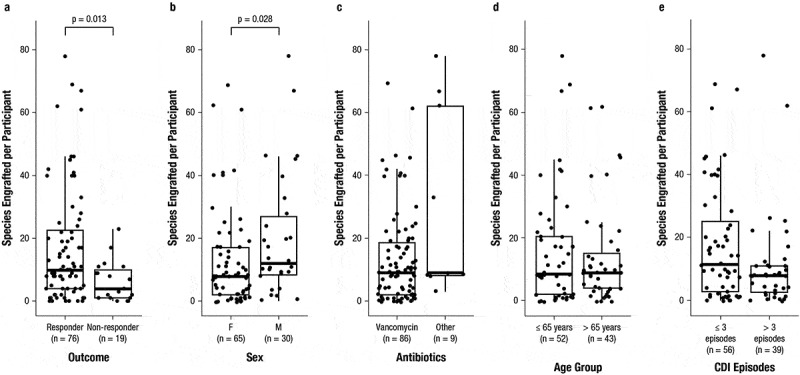
Engraftment among subgroups by a) outcome, b) sex, c) antibiotics used, d) age, and e) number of prior CDI episodes for participants administered RBL (*n* = 95). Median (thick horizontal lines) and interquartile ranges (boxes) are shown. CDI, *Clostridioides difficile* infection; F, female; M, male; RBL, fecal microbiota, live-jslm.

**Figure 6.**
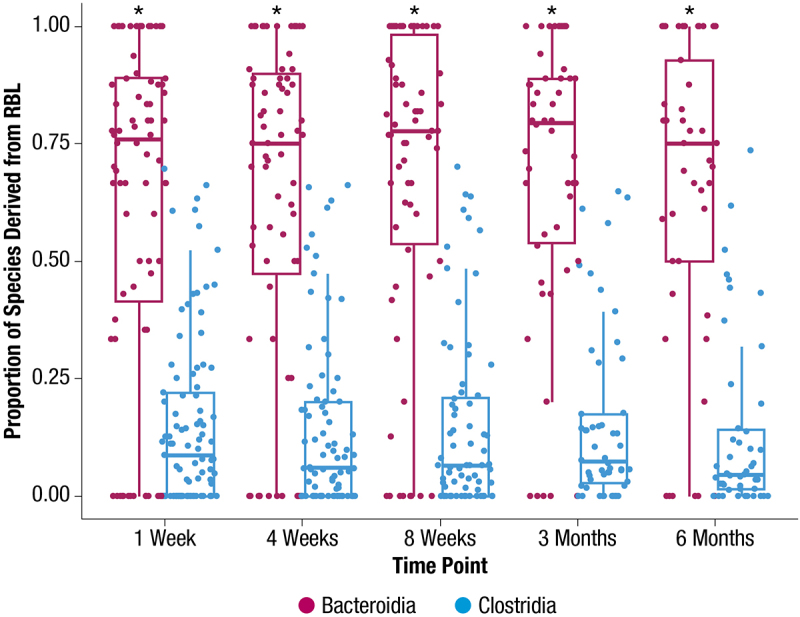
The fraction of species in post-administration RBL participant samples for which clonal engraftment from RBL was observed. Median (thick horizontal lines) and interquartile ranges (boxes) are shown. *p < 0.0001; RBL, fecal microbiota, live-jslm.

At 1 week after administration, there were significantly more engrafted RBL species among RBL responders (median of 10) than non-responders (median of 4; *p* = 0.013) (Figure 5(a)). There were also significantly more engrafted species in males compared with females at 1 week (*p* = 0.028) (Figure 5(b)). No significant differences in engraftment were observed between subgroups by antibiotic usage, age, or number of CDI episodes (Figure 5(c-e)). Among the detected species per participant after RBL administration, most Bacteroidia species were clonally engrafted from RBL, whereas only a minority of Clostridia species were engrafted from RBL (Figure 6).

### Engraftment effectiveness and bile acid gene composition

**Figure 7.**
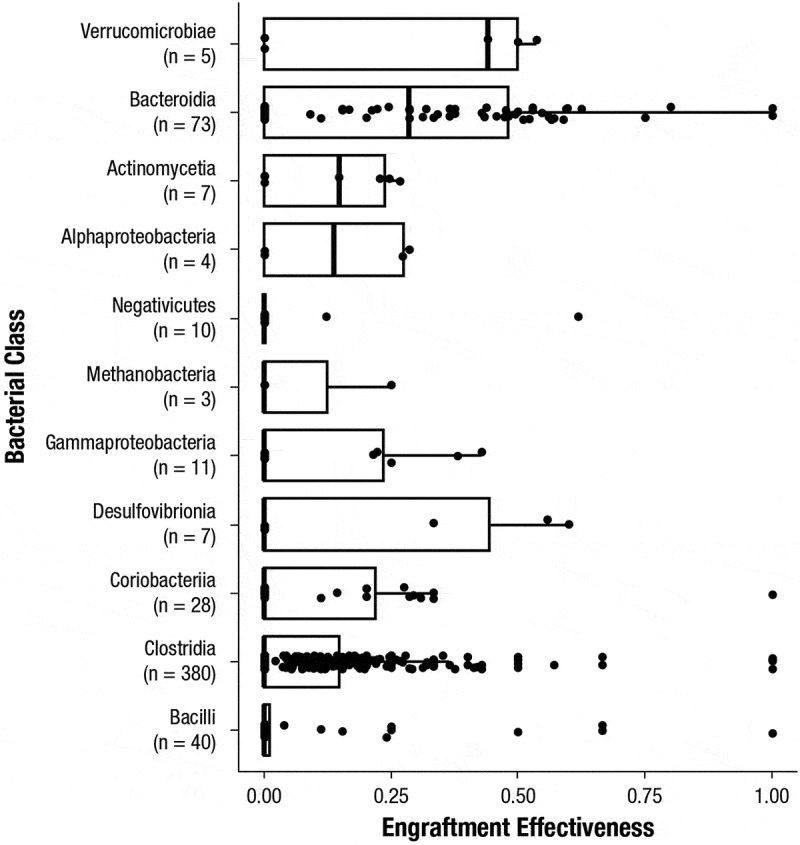
Engraftment effectiveness for all species within bacterial classes with ≥ 1 engraftment event detected. The median engraftment effectiveness per class is denoted by a heavy vertical line, with interquartile ranges denoted by boxes.

**Figure 8.**
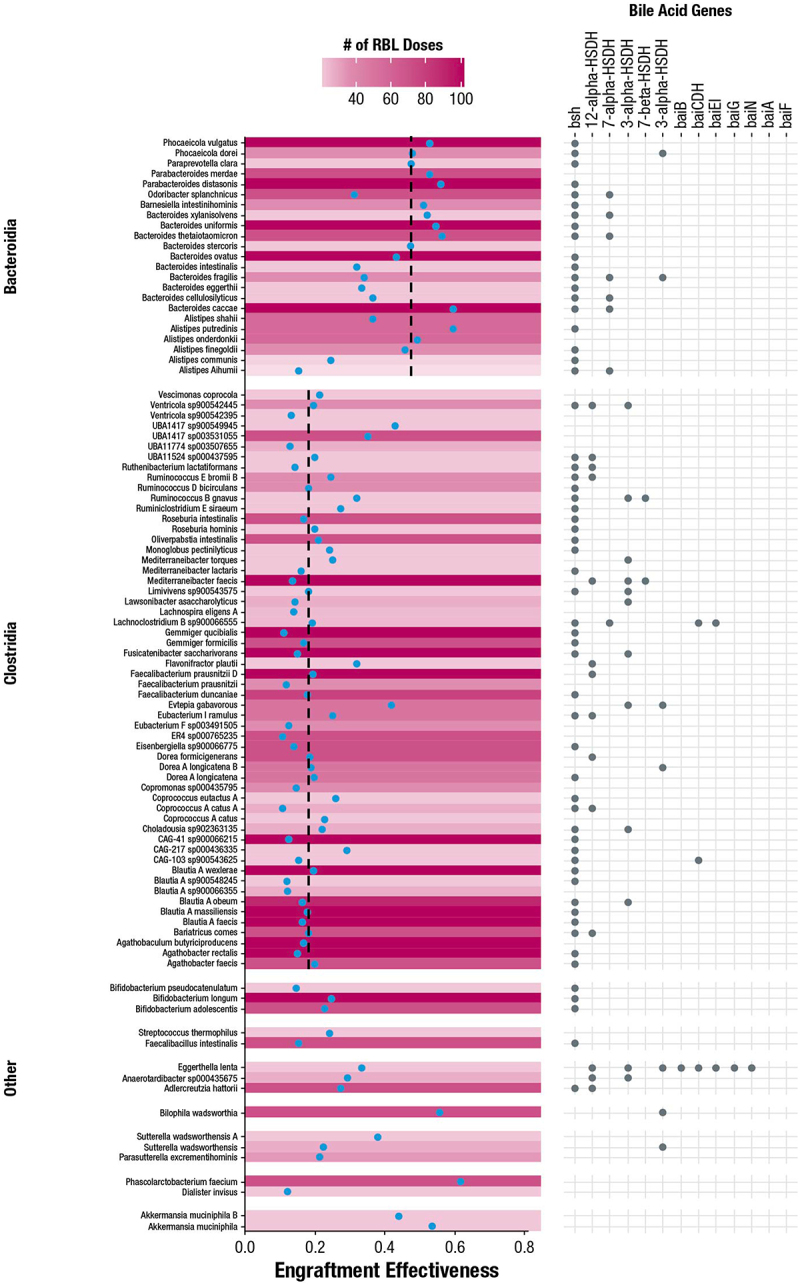
Per species engraftment effectiveness and bile acid gene composition in participants administered RBL. Blue dots represent the engraftment effectiveness for that species across RBL doses and participants, dashed lines represents the median engraftment effectiveness for species within that class of bacteria, gray dots signify the presence of the denoted gene within the reference genome for that species, and red gradient represents the number of RBL doses in which each species is found. Only species present in ≥ 20% of doses were analyzed. RBL, fecal microbiota, live-jslm.

Among taxonomic classes, 380 different Clostridia species and 73 different Bacteroidia species were found to engraft in ≥ 1 participant sample (Figure 7). Despite the large number of Clostridia species that engrafted, most had low engraftment effectiveness, which was defined as the number of participants in which engraftment was observed in ≥ 1 sample divided by the number of administered RBL doses which included that species. In contrast, Bacteroidia species had higher median engraftment effectiveness (median = 0.27; Figure 7). Among species detected in ≥ 20% of RBL doses, the most effective engrafters were *Phascolarctobacterium faecium* (engraftment effectiveness = 0.618), *Alistipes putredinis* (0.597), *Bacteroides caccae* (0.595), *Bacteroides thetaiotamicron* (0.565), and *Parabacteroides distasonis* (0.559) (Figure 8). Notably, the higher median engraftment effectiveness of Bacteroidia compared to Clostridia did not appear to be driven by the per-species average relative abundances in RBL (Supplementary Figure 4). The genomes of the most effective engrafter species are expected to include genes involved in bile acid metabolism, including bile salt hydrolase (*bsh*), which catalyzes the first step of primary to secondary bile acid conversion (Figure 8).^13,37^ Further, the number of engrafting species per participant correlated with the ratio of primary to secondary conjugated bile acids as reported in a previous analysis (Supplementary Figure 5).^25^

### Persistence of engrafted species over time

**Figure 9.**
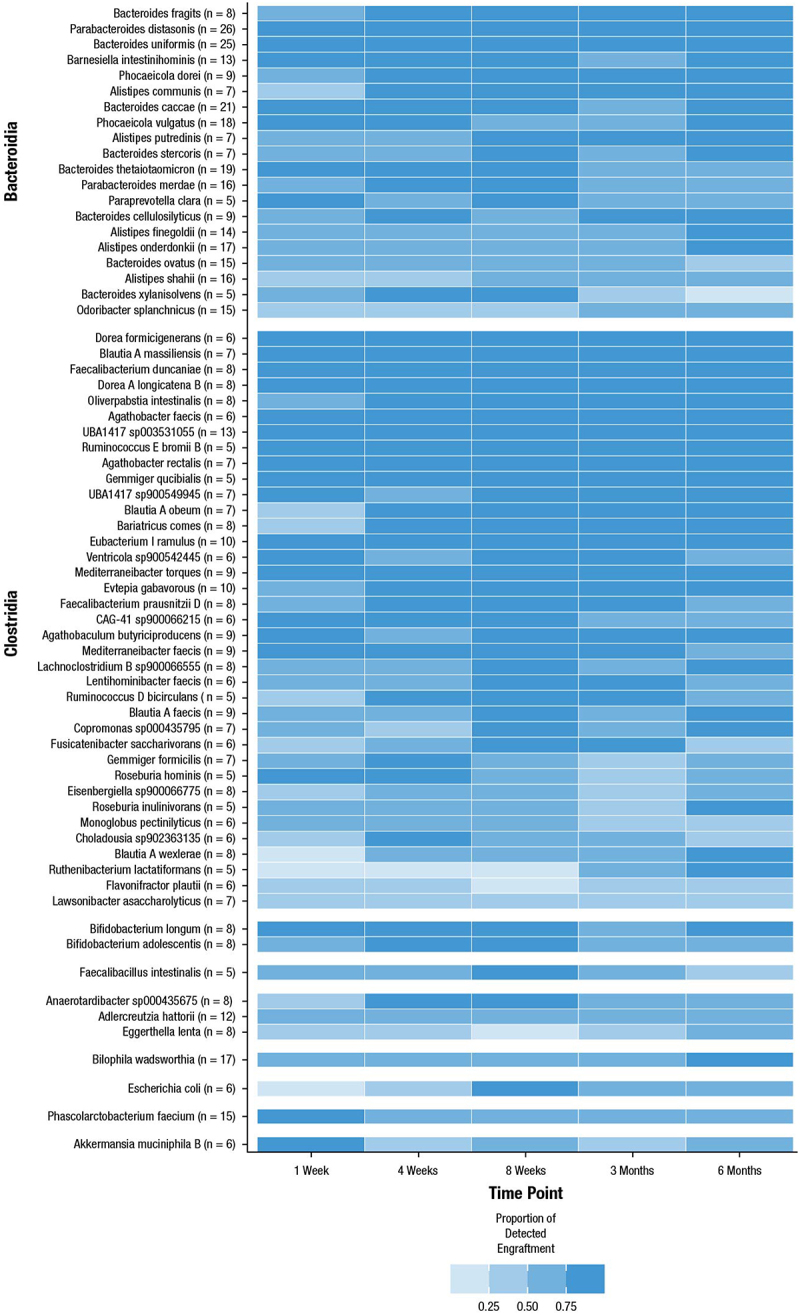
Persistence of per-species engraftment effectiveness over time for species engrafting in ≥ 5 participants. The number of participants with engrafting species is designated as n next to the species name with a maximum of *n* = 33. The blue gradient represents the proportion with engraftment detected for each species at each time point.

For each engrafting species, persistence was determined among the subset of participants who provided stool samples at all 5 requested time points after RBL administration (*n* = 33) and was defined as the number of participants at each time point in which clonal engraftment was observed divided by the number of participants in which clonal engraftment occurred at any time point. Engraftment persistence was generally high for most species (Figure 9), with several Bacteroidia, Clostridia, and Actinomycetia species engrafted at all time points. The 3 most persistent engrafters were *Parabacteroides distasonis* (*n* = 26/33 participants, persistence = 0.892 across all participants and time points), *Bacteroides uniformis* (*n* = 25/33 participants, persistence = 0.864), and *Bacteroides caccae* (*n* = 21/33, persistence = 0.79).

## Discussion

The recent approvals of 2 live biotherapeutics by the FDA are clear indications the gut microbiome can be a valuable target for modulating human health.^23,38^ Yet, fundamental drug development concepts such as PK, the distribution of a drug in the body over time, and PD, the physiologic and therapeutic effects of a drug, remain to be established for live biotherapeutics. So far, most microbiome or metabolome analyses that have been associated with clinical trials reported PD attributes – describing what changed or was restored after live biotherapeutic administration. Few studies have definitively examined disposition of the administered live biotherapeutic in the body after dosing, which is a PK attribute and which we term clonal engraftment. Herein, we leveraged deep metagenomic sequencing and a rigorous bioinformatic method to evaluate clonal engraftment of bacterial populations from RBL to participants. We propose that this approach could be applied in other programs as a more precise platform and definition for engraftment that would advance the development of PK/PD analyses of live biotherapeutics.

The term engraftment has been applied heterogeneously in the microbiome field, including defining it as convergence of recipient to donor alpha or beta diversity,^39^ increased number of live biotherapeutic species detected in recipients,^40^ or specific tracking of strains or clonal populations from therapeutic to recipients.^41^ For engraftment to be used as a PK descriptor, it should denote transfer and propagation of bacterial populations from an administered live biotherapeutic to patients, analogous to how the term is used in the stem cell transplant field. To discern engraftment, bioinformatic methods must have sufficient specificity to distinguish the origin of a population – the administered biotherapeutic versus environmental or pre-resident commensal populations. The specificity of species-level resolution is often insufficient; in a trial of a Clostridia-based biotherapeutic, species-level analyses showed significant increases in product-contained species even among placebo recipients.^40^ This made it unclear as to whether species changes in treatment recipients originated from the biotherapeutic or another source. In the present analysis, a trained weighted logistic regression method enabled highly specific resolution to a clonal population level, allowing identification of which strains had transferred from RBL to recipients. This specificity is evidenced by the negligible detection of apparent engraftment among placebo recipients (Figure 4). The method is also sensitive, as it could resolve RBL-derived clonal populations at a relative abundance as low as 0.1% on average, and in some cases lower (Supplementary Figure 3). Taken together, the high specificity and sensitivity of this method allows for a more precise analysis of engraftment of strains from biotherapeutics. In this study, engraftment analyses revealed a correlation between higher engraftment and clinical success (Figure 5(a)), which is a meaningful PK conclusion. Since deeper sequencing and more sophisticated bioinformatics tools are now available, it is recommended that future evaluations of microbiota-based products leverage engraftment analyses at strain or clonal population resolutions to enable better comparisons among different studies.

This refined engraftment framework allows deeper PK/PD questions to be assessed. In this study, Clostridia species were restored significantly even when Clostridia engraftment from RBL was low, indicating many of the restored species were not engrafted from RBL (Figure 6). Consistent with this, Clostridia were also significantly restored in placebo recipients, as has been observed in other trials.^40^ The capability of many Clostridia to sporulate may allow them to persist through CDI antibiotic treatment and thereby be poised for restoration, which has been observed in antibiotic-only trials.^42^ It has been suggested that spore-forming Clostridia are highly transmissible from environmental sources; therefore, it could also be that some Clostridia were restored from the environment rather than from RBL.^43^ Persistence through antibiotic treatment appears to be the more likely explanation, since our study also indicated Clostridia species had lower median engraftment effectiveness, which was also observed in studies of FMT^44^ and for a Clostridia-only live biotherapeutic.^41,45^ In these latter studies, a higher dose^40^ or multiple doses^36^ of the biotherapeutic had to be administered for 14 days to achieve engraftment of a majority of the product strains. The mechanisms of lower Clostridia engraftment effectiveness are not clear but may be due to nutritional competition between resident Clostridia that persisted through antibiotic treatment and product-derived Clostridia, a model that has been suggested to underpin *C. difficile* colonization resistance by healthy microbiota.^46^ The lack of selective pressure on Clostridia to effectively engraft has been suggested to explain the inverse correlation between their engraftment effectiveness and spore-forming capability.^44^ Collectively, our data indicate that successful engineering of Clostridia-based biotherapeutics could benefit from rational selection of more effective engrafters.

Unlike Clostridia, Bacteroidia restoration in this study appeared to be driven by engraftment since the majority of restored Bacteroidia species were engrafted from RBL (Figure 6) and since Bacteroidia restoration was low among placebo recipients (Figure 2). Poor restoration among placebo recipients is consistent with slow Bacteroidia restoration after CDI antibiotic treatment alone,^42,47^ and higher restoration among RBL recipients is consistent with high engraftment effectiveness of Bacteroidia species (Figures 7 and 8), which has also been seen after FMT.^48^ It may be that many Bacteroidia species have evolved metabolic functions that contribute to their higher engraftment effectiveness, such as pathways for metabolizing O-glycans^49^ and mucins.^50,51^ CDI antibiotic treatment may also largely deplete Bacteroidia from recipients, resulting in a less competitive environment for RBL-derived Bacteroidia. Collectively, our data indicate engraftment of RBL-derived Bacteroidia drove Bacteroidia restoration in this study, and the generally high engraftment potential of many Bacteroidia species makes them well suited pharmacokinetically for inclusion in microbiota-based products, provided they have the appropriate functions for eliciting efficacy.

This study also revealed that many clonal populations that engrafted after RBL administration persisted for at least 6 months (Figure 9). Persistence of engraftment was also observed in FMT studies, with some species detectable after 5 years or more.^26^ This temporal understanding of the distribution of drug ingredients is fundamental to PK analysis of a therapeutic. Notably, persistence did not appear to differ among bacterial classes, which suggests that persistence may be determined by initial engraftment effectiveness, rather than being attributable to class-specific functions. Once successfully engrafted, community stability may encourage persistence, as has been suggested by ecological modeling of microbiome community dynamics and mutualistic interactions.^52^ In our study, the persistence of engraftment and community stability may underpin the sustained clinical response (lack of rCDI recurrence) through 6 months after RBL administration.^16^ Microbiome restoration and sustained clinical response were also observed out to 24 months in an earlier trial of RBL.^53^ Our analyses therefore indicate microbiota-based products such as RBL have the potential to engraft and induce long-term microbiome restoration that could have durable clinical impacts.

The large number of RBL doses, participants, and clonal populations in this study strengthened the conclusions that could be made about engraftment. For example, the finding that the Bacteroidia species *Phocaeicola vulgatus* had high engraftment effectiveness (Figure 8) is based on data from 102 administered doses of RBL. Further, since those doses were from 27 different donors, they likely represented different strains or clonal populations, suggesting the generality of conclusions about that species’ engraftment. In turn, analysis of the large collection of RBL doses supported a general conclusion that most RBL doses contained moderately to highly effective engrafters (Figure 8). Having multiple clonal populations with high engraftment potential may be a strength of broad-consortium microbiota-based products such as RBL, particularly if engraftment is significantly influenced by host factors that vary among patients, as has been suggested.^41,45,54^ The analyses herein also indicated many of the moderate to high engrafting species have genes that support bile acid metabolism (Figure 8), including Bacteroidia species previously reported to exhibit the highest primary:secondary bile acid transformation activity,^55^ and thus the likelihood of engrafting these functions was high for each RBL recipient. This may explain the association of higher engraftment with higher bile acid conversion (Supplementary Figure 5), as well as the increased bile acid conversion among RBL responders compared with placebo responders.^25^ The observed correlation between engraftment of bile acid‒metabolizing species and changes in bile acid production is an example of the interplay between PK and PD, and these understandings are important for determining mechanisms of action for microbiota-based products and guiding their future development.

A potential methodological limitation of this study is the use of rarefaction prior to diversity analyses, which may result in data loss,^56,57^ although it should be noted that engraftment findings were based on non-rarefied data. Additionally, the results are reported at the species level, as this approach does not track multiple strains per species from an individual participant which would provide increased resolution into engraftment from RBL doses. Several questions remain to be answered for a full understanding of the PD effects of RBL clonal population engraftment. This study did not aim to determine whether or how the engrafted species affected the rest of the restored microbiome composition (e.g. non-engrafted Clostridia), or whether specific engrafters helped eliminate Gammaproteobacteria and Bacilli, as has been proposed for FMT.^58^ These hypotheses will be explored in future studies. In addition, because this analysis was exploratory, future prospective studies are needed to definitively conclude which engrafters, or their metabolic capacity, are driving efficacy in prevention of rCDI. Similarly, although per-species engraftment of functional genes is suggested in the outcomes of this analysis, direct quantification of such genes is planned for future studies to strengthen those conclusions. Finally, to reach a fully defined framework for describing RBL PK, whole-genome assembly of engrafting strains and determination of their associated relative abundances over time will be required, which will also facilitate advanced modeling of growth kinetics after engraftment. This study advances the ability to answer these questions by providing a quantitatively rigorous method for assessing microbiota-based product strain engraftment into patients, illuminating possible mechanisms by which RBL prevents rCDI, and progressing the framework for describing the PK and PD of live biotherapeutics.

## Supplementary Material

Engraftment MS_Supplementary Materials_Resubmission_FINAL.docx

## Data Availability

Ferring Pharmaceuticals, Inc., will provide access to individual de-identified participant data upon request via a secure portal, to researchers whose proposals meet the research criteria and other conditions. To gain access, data requestors must enter into a data access agreement with Ferring.
