# Supplementary material for: Microbiome compositional changes and clonal engraftment in a phase 3 trial of fecal microbiota, live-jslm for recurrent Clostridioides difficile infection
Source: Gut Microbes. 2025 Jun 24;17(1):2520412. doi: 10.1080/19490976.2025.2520412 (PMC12931727; doi:10.1080/19490976.2025.2520412)
Supplement: Engraftment MS_Supplementary Materials_Resubmission_FINAL.docx [file KGMI_A_2520412_SM7055.docx]

# **Microbiome compositional changes and clonal engraftment in a phase 3 trial of fecal microbiota, live-jslm for recurrent *Clostridioides difficile* infection**

# **Authors:** Josh Claypool,^a^ Gustav Lindved,^b^ Pernille Neve Myers,^b^ Tonya Ward,^a^ Henrik Bjørn Nielsen,^b^ Ken F. Blount^a*^

# **Supplementary Materials**

# **Supplementary Figures**


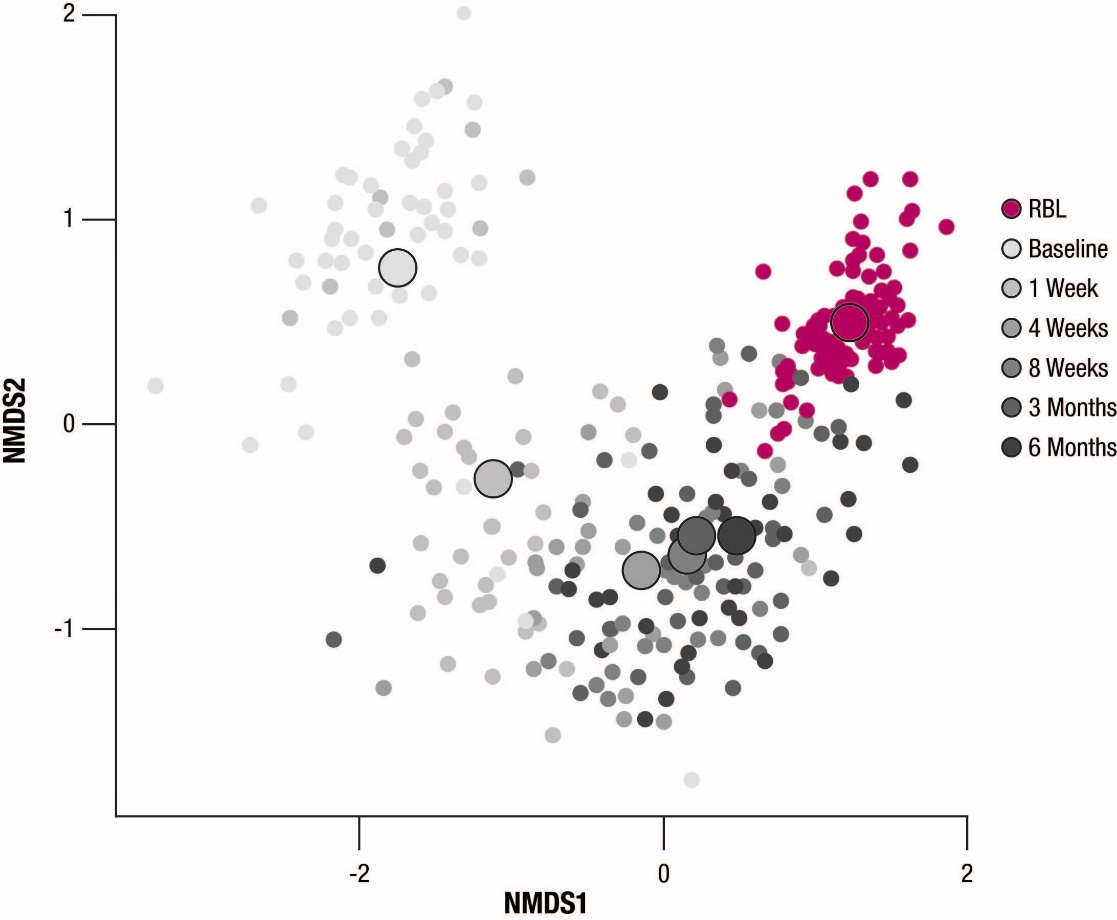


**Supplementary Figure 1**. NMDS of Bray-Curtis distance calculated on species-level taxonomy of stool samples for baseline (n = 49 participants) and post-placebo administration time points (n = 49 participants). NMDS, non-metric multidimensional scaling; RBL, fecal microbiota, live-jslm.


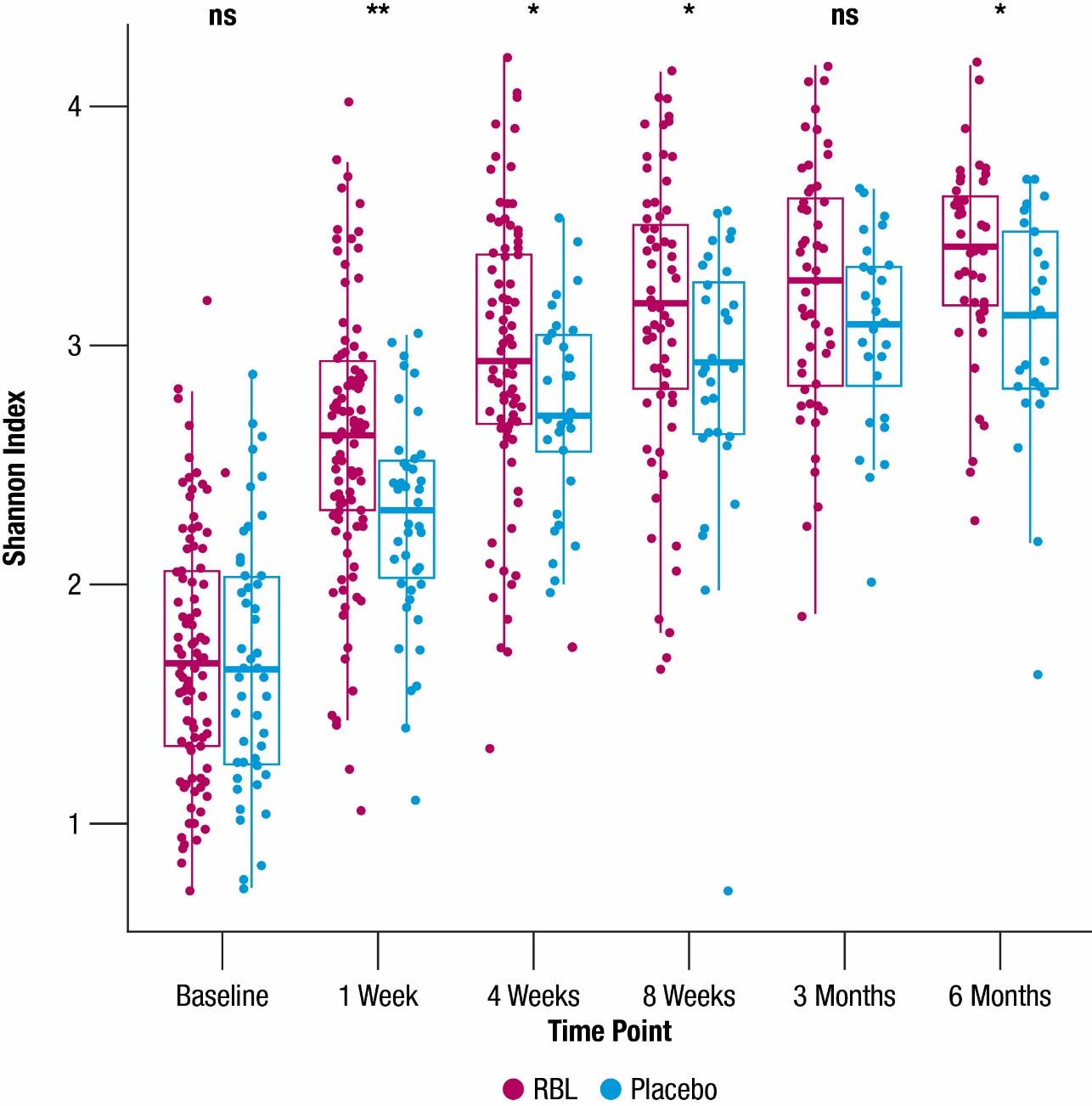


**Supplementary Figure 2.** Shannon Index following RBL and placebo administration. Significance assessed via Wilcoxon rank-sum test. ns = not significant, *p < 0.05, **p < 0.001. RBL, fecal microbiota, live-jslm.


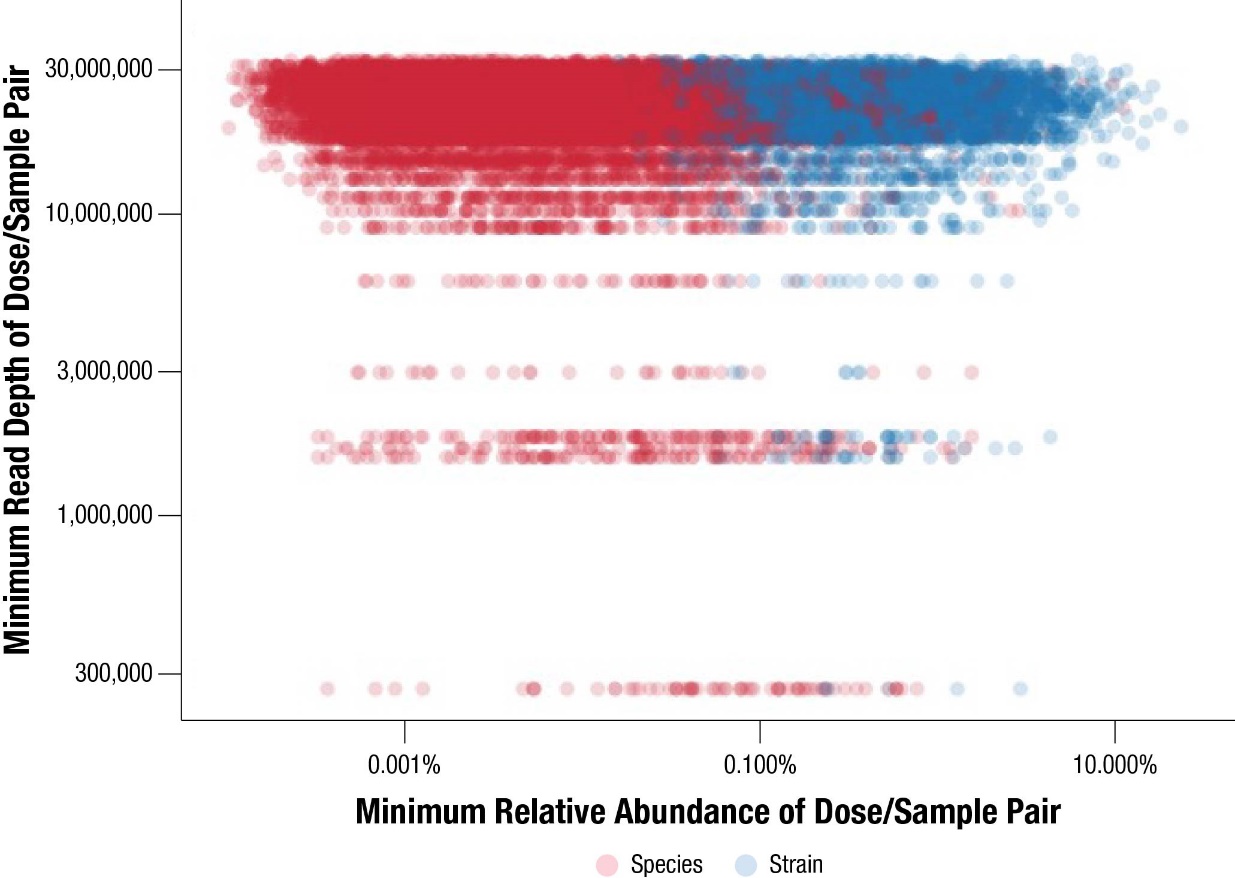


**Supplementary Figure 3.** The smallest read depth of dose-sample pairs (y-axis) and minimum relative abundance of species (x-axis) within each dose-sample pair. Colors represent if a species was evaluated for engraftment (blue) or not (red) within each dose-sample pair.


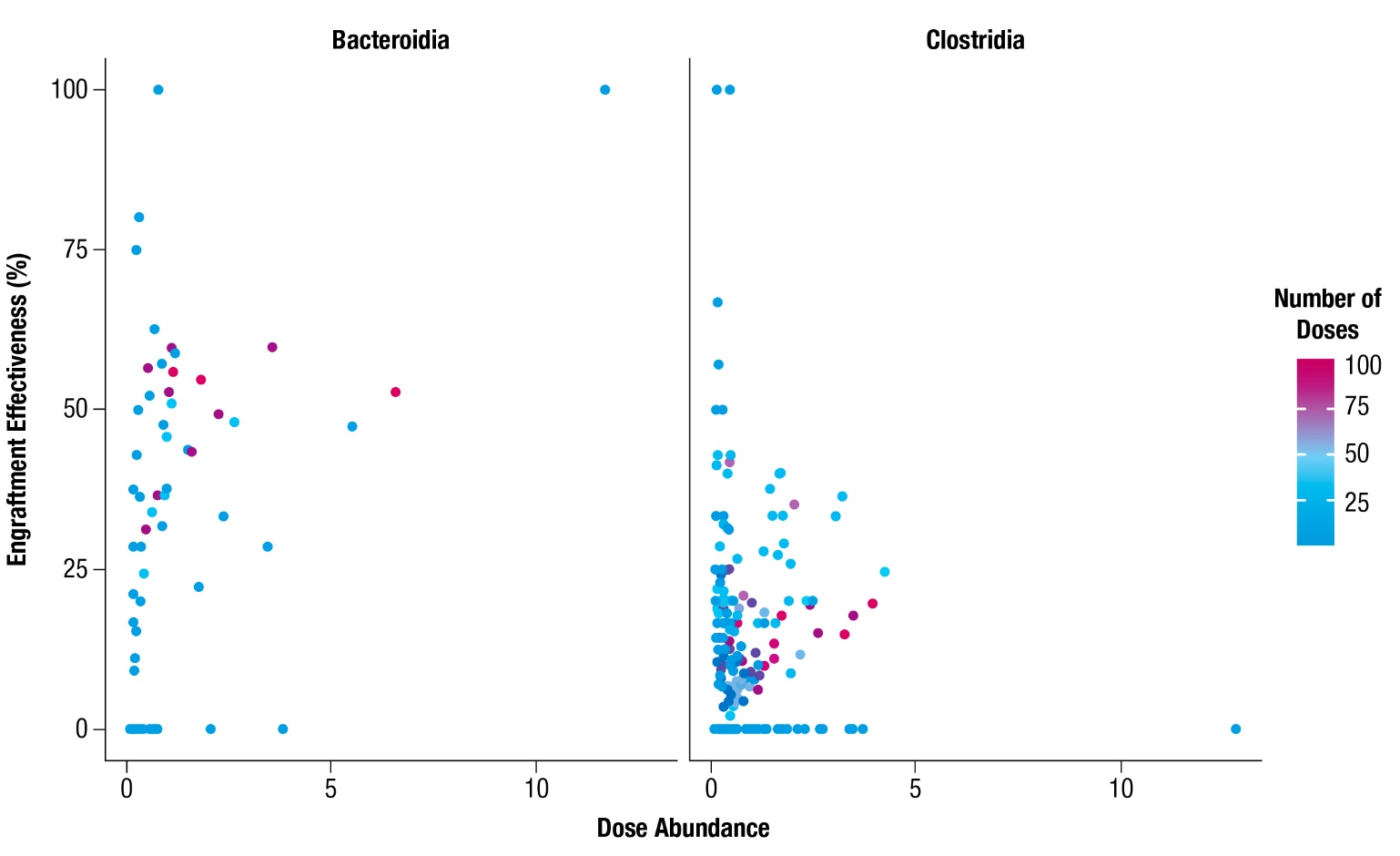


# **Supplementary Figure 4.** Engraftment effectiveness of Bacteroidia and Clostridia species as a function of the average per-species relative abundance in RBL doses where the species was detected. Species are color coded by the number of RBL doses in which each were identified. RBL, fecal microbiota, live-jslm.

**
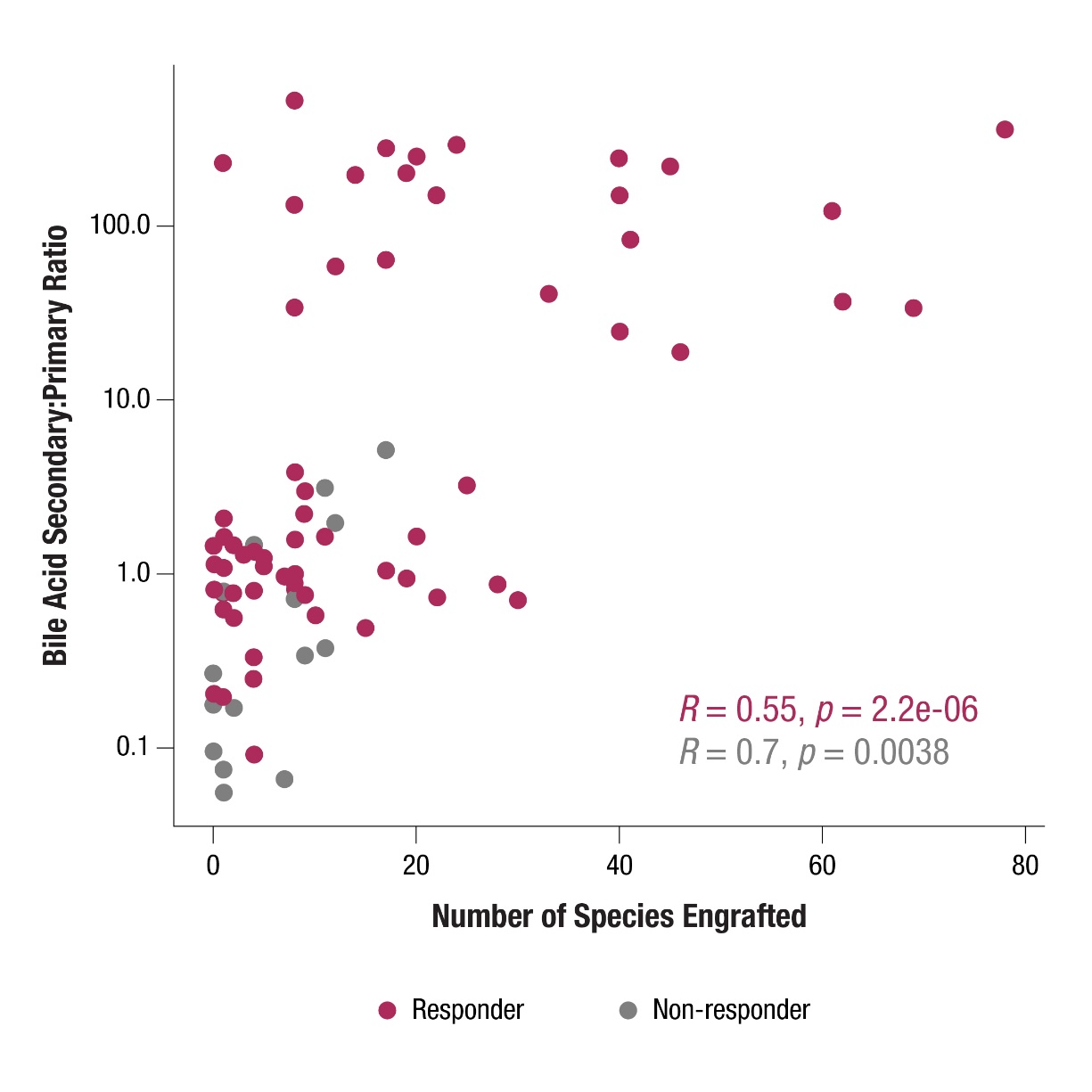
**

# **Supplementary Figure 5.** The ratio of stool secondary:primary bile acids per participant administered RBL at 1 week (N = 80) plotted according to the respective number of species engrafted. Participants are colored by their outcome (responder = 65, non-responder = 15). Pearson correlation coefficients were determined between the number of engrafted species and ratio of secondary to primary bile acids for responders and non-responders. RBL, fecal microbiota, live-jslm.
